# Traditional Dual Growing Rod Technique in the Management of Early Onset Scoliosis and Its Effects on Spinal Growth and Lung Development: The Mid-Term Prospective Results

**DOI:** 10.7759/cureus.14422

**Published:** 2021-04-11

**Authors:** Bertan Cengiz, Haci Mustafa Ozdemir, Abdurrahman Sakaogullari, Metin Isik, Nevres Hürriyet Aydoğan

**Affiliations:** 1 Orthopedics and Traumatology, Acibadem Kayseri Hospital, Kayseri, TUR; 2 Orthopedics and Traumatology, Sisli Hamidiye Etfal Training and Research Hospital, Istanbul, TUR; 3 Orthopedics and Traumatology, Ankara Training and Research Hospital, Ankara, TUR; 4 Orthopedics and Traumatology, Mugla Sitki Kocman University, Muğla, TUR

**Keywords:** early onset scoliosis, dual traditional growing rod, space available for lung, complications

## Abstract

Objective: The purpose of this study was to investigate the safety and effectiveness of the traditional dual growing rod (TDGR) technique, using only pedicle screws for fixation with more frequent lengthening while evaluating scoliosis correction in the growing spine, spinal growth rates, and the differences in lung volumes.

Patients and methods: In this single-centre prospective study, 27 patients with a follow-up of over three years were included in the study. Only pedicle screws were used as foundations for fixation. Routine lengthening procedures were performed every six months. Data were recorded including the age of initial surgery, gender, number of lengthenings, follow-up, and complications. The Cobb angle of the major curve, kyphosis angle, T1- S1 length, space available for lung (SAL) ratio, coronal and sagittal balance, and the height of all patients were measured and recorded preoperatively, immediately postoperatively, and finally before and after every lengthening.

Results: The average follow-up time was 46.3 months (36-64 months). The correction rate was 69.5% for Cobb angle and 43.2% for kyphosis between preoperative and final follow-up period. The time between two lengthenings was 6.9 months, and the mean T1-S1 length increase was 1.78 cm per year. The SAL ratio increased from 0.885 preinitially to 0.985 at the last follow-up. The complication rate was determined as 9.6% in 187 procedures. Acceptable improvements were determined in the specified parameters with low complication rates with the use of this technique.

Conclusion: The TDGR technique with proximal and distal pedicle screws as anchors is a safe and effective treatment for deformity control in selected patients with early onset scoliosis (EOS). Repetitive surgical interventions are the negative side of this technique.

## Introduction

Treatment of early onset scoliosis (EOS) is one of the most difficult challenges in pediatric spine surgery. Early spinal fusion affects the spinal column and cardiopulmonary system and may lead to a shortened trunk and thoracic insufficiency syndrome (TIS) owing to a diminished thoracic cavity [1]. Spinal instrumentation without fusion, including the use of traditional growing rods (TGR), has been suggested as a modality that allows for spinal growth in the treatment of EOS [2]. Improvements in results for the treatment of progressive EOS have been reported from the dual TGR (DTGR) technique when compared with the single rod technique [3].

All of these growth sparing systems include rods connected with different types of proximal and distal anchors as pedicle screws or hooks. The most significant difference between these anchors is pull-out strength. Biomechanical comparisons of different anchors for the TGR technique have shown that pedicle screws have been associated with greater pull-out strength [4].

This prospective study evaluated children treated with TGR surgery in our institution. The aim of the study was to investigate the safety and effectiveness of the DTGR technique using pedicle screws as anchors in the proximal and distal foundations while evaluating scoliosis correction in the growing spine, spinal balance, spinal growth rates, and the differences in lung volumes.

## Materials and methods

In this prospective study, 31 patients (14 male, 17 female) were diagnosed with EOS and instrumented with the DTGR technique without fusion between 2010 and 2013. Surgical indications to initiate growing rod treatment were Cobb angle >40°, curve progression >10° on repeated radiographs, or severe kyphotic deformity angle of >80° with no response to conservative treatment. Patients followed-up for less than three years after surgery and patients older than 10 years old at index surgery were removed from the study. Twenty-seven of these 31 patients met the criteria and were included in the study. Radiological and physical measurements of the patients were made by two separate observers (B.C. and M.I.) before and after every procedure and the last follow-up examination, and the average of these measurements was recorded.

The study was approved by the ethics committee of our institute and written informed consent - for the procedures and also the use of data as part of a scientific study - was obtained from all patients or their caregivers.

Surgical technique

The surgical procedure used in all the study patients was the DGR technique as described by Akbarnia et al. [5]. The index surgery was performed through two midline incisions, at the level of the proximal and distal foundations which were preoperatively planned, based on the type and location of the curve. The exposure was sub-periosteal only at the levels of the foundations to avoid spontaneous fusion. After confirmation of the vertebral level with the image intensifier, pedicle screws were instrumented as proximal and distal anchors, a minimum of four pedicle screws were used. Only pedicle screws were used to achieve greater pull-out strength and no hooks were used in the index surgery as anchors. To secure the foundation anchors, fusion was achieved by decortication and the application of allograft to augment bony fusion. After contouring the rods for sagittal alignment, the rods were placed subcutaneously and connected with tandem connectors placed at the thoracolumbar junction and no cross-connectors were used. Following index surgery, the lengthening procedure was performed regularly every six months. There was no routine use of a postoperative brace or routine spinal cord monitoring. All procedures (index surgery and distractions) were performed by two experienced spine surgeons (H.M.O. and A.S.).

 Data analysis

The gender, age, diagnosis of patients, and surgical information (levels of instrumentation, number of pedicle screws used, lengthening intervals, and number of lengthenings) were recorded. Standing full spine x-rays were taken before and after the index surgery and every lengthening procedure. Radiographs were taken in the coronal and sagittal planes to assess scoliosis, kyphosis, coronal and sagittal balance, and the space available for lung (SAL) index. The Cobb method was used to measure scoliosis degree. T1-S1 length was measured with a scanogram and standing heights were measured before and after index surgery and at the final follow-up examination. Two observers measured each radiograph independently. All surgical complications were recorded. Changes in radiological findings and height measurements between preoperative, postoperative, and follow-up periods were tested using the paired samples t-test. All analyses were performed on SPSS version 21 (IBM Corp., Armonk, NY, USA). A value of p<0.05 was considered statistically significant.

## Results

**Figure 1:**
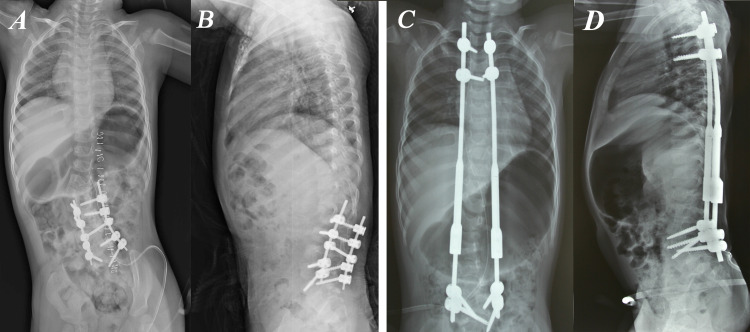
Photographs of a two-year-old girl with congenital scoliosis with hemivertebrae. The only patient that had traditional dual growing rod (TDGR) applied as a revision surgery in our study. Correction and short-segment posterior instrumentation was applied in a different center before. (A) Posteroanterior (PA) and (B) lateral X-Rays of the initial surgery. Decompensation had occurred when they applied to our clinic. (C) PA and (D) lateral X-Rays after TDGR surgery. TDGR was applied between T2-L5 and correction was achieved in this way.

The study included a total of 27 patients, comprising 11 males and 16 females, who underwent TDGR surgery. The patient diagnoses were as follows: 10 juvenile, two infantile, 14 congenital, and one neuromuscular scoliosis. Of these patients, surgical decompression of spur was applied to only one patient before GR surgery. GR surgery was applied as primary surgery for 26 of 27 patients for the deformity and was applied as revision surgery to only one patient who had received correction and short-segment posterior instrumentation but decompensation developed (Figure 1).

The mean age at surgery was 5.5 years (range 1.9-8.8 years). The average follow-up time was 46.3 months (range 36-64 months). The mean number of lengthenings was 5.8 (range 3-7 lengthenings) per patient. The average time between two lengthenings was 6.9 months (range 6-9.3 months). Final fusions were performed for four patients (14.8%) at the time of the study. The mean age of the patients at the time of final fusion was 12.9 years. Of the 187 procedures performed within the treatment period, 27 (14.4%) were index surgery, 156 (83.5%) were lengthenings and four (2.1%) were final fusion surgery (Table 1).

**Table 1:** Patient demographics M: Male, F: Female, S: Scoliosis, mo: months

Patient number	Age (year)	Gender	Diagnosis	Follow-up (mo)	Number of lengthenings	Lengthening interval (mo)	Final Fusion Time (age)
1	8,2	F	JUVENILE IDIOPATHIC S	64	6	7,9	12,8
2	8,8	F	JUVENILE IDIOPATHIC S	57	6	6,2	13,2
3	6,6	F	JUVENILE IDIOPATHIC S	56	6	7,9	
4	2,4	M	CONGENITAL S	54	7	6,8	
5	2,6	F	CONGENITAL S	54	7	6,8	
6	8,7	F	JUVENILE IDIOPATHIC S	53	6	7,5	13
7	6,3	F	NEUROMUSCULAR S	53	7	6,8	
8	3,3	M	INFANTILE IDIOPATHIC S	48	7	6,1	
9	4,3	F	CONGENITAL S	48	7	6,2	
10	7,1	F	CONGENITAL S	48	6	7	
11	3,3	M	CONGENITAL S	47	6	7	
12	5	M	INFANTILE IDIOPATHIC S	47	6	6,9	
13	5,4	M	JUVENILE IDIOPATHIC S	46	7	6	
14	8,3	M	JUVENILE IDIOPATHIC S	46	5	6,8	12,6
15	2	F	CONGENITAL S	46	7	6	
16	7,1	F	JUVENILE IDIOPATHIC S	45	6	6,5	
17	7,8	F	JUVENILE IDIOPATHIC S	44	6	6,6	
18	4,1	F	CONGENITAL S	44	5	7,4	
19	6,1	F	CONGENITAL S	44	5	7,6	
20	1,9	M	CONGENITAL S	41	5	7	
21	1,8	F	CONGENITAL S	40	6	6,2	
22	5,6	F	JUVENILE IDIOPATHIC S	40	6	6	
23	8,8	M	CONGENITAL S	39	5	6,4	
24	6	M	CONGENITAL S	38	5	6,2	
25	6,6	F	CONGENITAL S	36	3	9,3	
26	8	F	JUVENILE IDIOPATHIC S	36	4	8,2	
27	2	M	CONGENITAL S	36	4	8	

**Figure 2:**
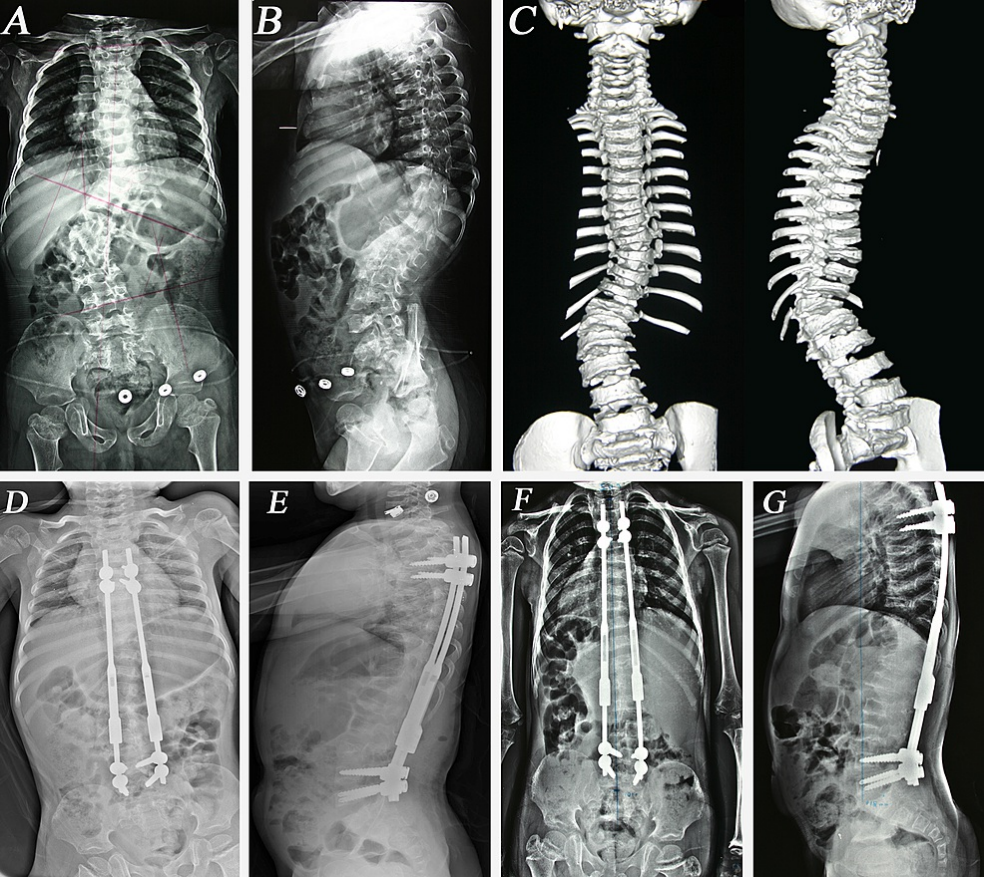
The course of treatment of a patient with congenital scoliosis Female, 2.6 y/o, congenital scoliosis. Thoracic curve 24°, thoracolumbar curve 52° and kyphosis 66°, T1- S1 height 217 mm. (A) PA, (B) lateral X-Rays and (C) 3D CT before initial surgery. (D) Posteroanterior (PA) and (E) lateral X-Rays after initial surgery, thoracic curve 12°, thoracolumbar curve 20°, kyphosis 44°, T1-S1 height 255 mm. (F) PA, (G) lateral X-Rays after 7th lengthening; thoracic curve 6°, thoracolumbar curve 16°, kyphosis 36° and T1- S1 length 286 mm.

The major Cobb angle was 53.7° (34°-86°) preinitially, 22.6° (0°-46°) postinitially (p<0.05) and 16.4° (0°-36°) at the final follow-up (p<0.05). Correction in the major Cobb angle was 57.9% in the postinitial period and 69.5% at the final follow-up. The kyphosis angle (T2-12) was measured as 48.4° (6°-98°) preinitially, 26.9° (8°-66°) postinitially (p<0.05) and 27.5° (10°-60°) at the final follow-up (p=0.496). Correction in the kyphosis angle was 44.4% at the postinitial period and 43.2% at the final follow-up. The mean T1-S1 length was 250.4 mm (173-330) preinitially, 281.2 mm (198-362) postinitially (p<0.05) and 319.2 mm (222-389) at the final follow-up (p<0.05). The mean T1-S1 length increase was 1.78 cm per year (Figure 2). The standing height was measured as 105.4 cm (70.5-142) preinitially, 107.4 cm (72.5-144.5) postinitially (p<0.05) and 119.6 cm (83.5-158) at the final follow-up (p< 0.05). The standing height increased by 2 cm (1.5-6) at the postinitial period and 12.2 cm (4-43) at the final follow-up. The SAL ratio increased from 0.885 (0.773-1) preinitially, to 0.956 (0.842-1.066) postinitially (p<0.05) and to 0.985 (0.825-1.092) at the final follow up (p<0.05) (Tables 2, 3).

**Table 2:** Patient data about results of correction, growth of the spine, change in the SAL ratio. SAL: space available for lung

Patient	Preinitial Cobb	Postinitial Cobb	Final Cobb	Preinitial Kyphosis	Postinitial Kyphosis	Final Kyphosis	Preinitial T1-S1 (mm)	Postinitial T1-S1 (mm)	Final T1-S1 (mm)	Preinitial SAL Ratio	Postinitial SAL Ratio	Final SAL Ratio
1	35	14	4	38	28	30	288	312	360	0.952	0.973	1
2	44	10	6	58	25	24	312	334	374	0.941	1	1
3	70	30	24	58	46	50	288	317	342	0.859	0.93	0.969
4	34	27	12	32	23	20	184	233	279	0.878	0.901	0.979
5	52	20	16	66	44	36	217	255	286	0.812	0.842	0.952
6	36	10	4	32	28	30	321	362	389	0.909	0.988	1.043
7	70	16	6	22	27	20	261	320	346	0.8	0.923	1
8	36	16	6	82	30	44	228	253	314	0.933	0.945	0.984
9	40	12	10	8	12	12	244	256	316	0.9	1	1
10	38	14	8	64	42	42	257	286	330	0.914	1	1
11	82	22	20	98	66	60	228	242	288	0.923	0.938	0.983
12	60	46	26	40	18	20	242	280	315	0.917	1.066	1.067
13	42	12	6	26	22	24	250	286	309	0.919	1	1
14	66	20	8	6	10	12	317	348	376	0.973	1	1.056
15	50	18	24	44	24	24	193	258	284	1	1.042	1.092
16	44	18	14	44	26	34	271	301	335	0.963	1	1
17	74	36	36	30	34	34	232	263	318	0.8	1	1
18	52	38	26	28	18	14	215	225	283	0.817	0.854	0.867
19	86	26	22	90	20	20	265	300	322	0.816	0.923	0.964
20	46	24	24	50	34	32	198	210	242	0.8	0.937	0.981
21	58	32	24	30	14	16	173	198	222	0.773	0.844	0.823
22	68	32	22	80	26	24	255	310	354	0.915	0.947	0.967
23	38	15	14	63	16	18	330	348	378	0.972	0.973	0.976
24	58	26	24	64	24	22	234	256	308	0.858	0.922	0.942
25	72	30	24	56	16	26	256	278	310	0.779	0.949	0.975
26	46	20	18	20	8	10	310	330	359	0.927	0.964	0.979
27	36	28	25	16	20	20	195	209	244	0.851	0.892	0.902

**Table 3:** Radiographic data of the patients preinital, postinitial and the last follow-up. SAL: space available for lung

	Preinitial	Postinitial	Final
Scoliosis (^0^)	53.7^0^ (34^0^-86^0^)	22.6^0^ (10^0^-46^0^)	16.4^0^ (4^0^-36^0^)
Thoracic Kyphosis (^0^)	48.4^0^ (6^0^-98^0^)	26.9^0^ (8^0^-66^0^)	27.5^0^ (10^0^-60^0^)
T1-S1 (mm)	250.4 (173-330)	281.2 (198-362)	319.2 (222-389)
SAL Ratio	0.885 (0.773-1)	0.956 (0.842-1.066)	0.985 (0.825-1.092)
Coronal Balance (mm)	15.4 (0-30)	9.8 (0-30)	9.6 (0-30)
Sagittal Balance (mm)	-7.5 (-84-60)	-2.1 (-52-40)	-5.8 (-60-60)

The average coronal balance was measured as 15.4 mm (0-30) preoperatively, decreased to 9.8 mm (0-30) at the postinitial period (p<0.05), and decreased to 9.6 (0-30) mm at the final follow-up (p>0.05). The average sagittal balance was measured as -7.5 mm (-84-60) preoperatively, -2.1 mm (-52-40) at the postinitial period (p>0.05) and -5.8 mm (-60-60) at the final follow-up (p>0.05) (Table 3).

**Figure 3:**
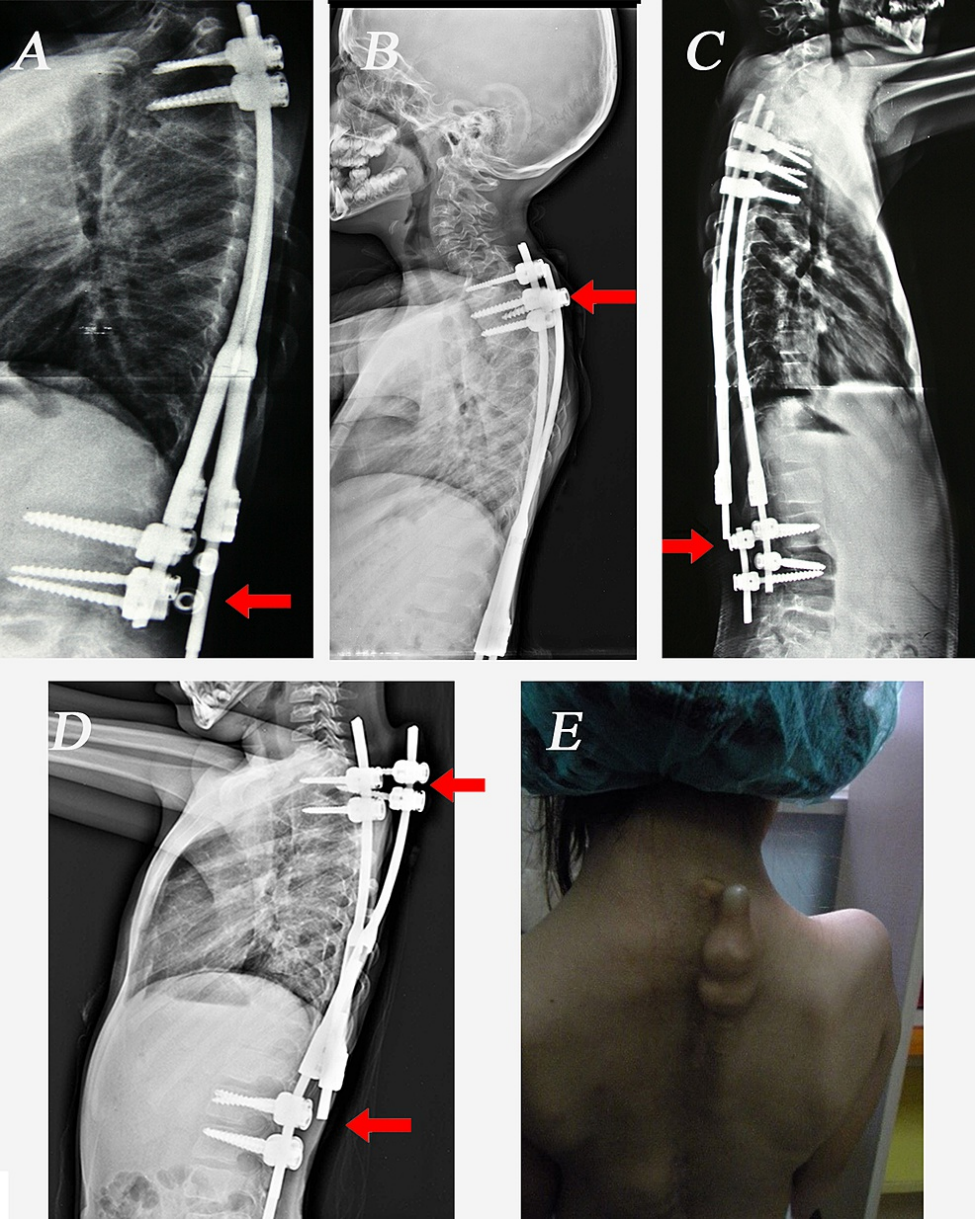
The implant-related complications in our patients. (A) End cap loosening and rod dislodgement, (B) Pedicle screw pullout, (C) Rod breakage (D) Rod breakage with pedicle screw pullout. (E) Photograph of a patient with proximal pedicle screw pullout

There were a total of 18 (9.6%) complications in 187 procedures. Superficial wound infection occurred in eight patients. No deep infection occurred in any patient throughout the follow-up period. There were 10 implant-related complications: three rod breakage, two screw pullout, one rod breakage with screw pullout, and four end cap loosening (Figure 3). No correction loss occurred after these complications. Four unplanned operations were performed for these complications. No neurological complications were encountered.

Pedicle screws were used as anchors in both the proximal and distal foundations for all patients at index surgery. The vertebral levels most used for screw placement at the proximal foundation were T3 (19.1%) and T4 (18.6%) and at the distal foundation, L3 (16.2%) and L4 (19.1%). The growing rods were connected end-to-end in 26 patients with tandem connectors, while they were connected side-to-side in only one patient. Transverse connectors were not used in any patient.

## Discussion

Scoliosis is a three-dimensional deformity of the spine and may reduce the thoracic volume that may lead to respiratory failure. EOS patients may be at risk of TIS (thoracic insufficiency syndrome) because of thoracic deformities secondary to scoliosis. In cases of EOS left untreated, there has been reported to be an increased risk of mortality due to respiratory failure [6]. Scoliosis will impair the development of alveolus and pulmonary arteriole due to thoracic deformities in children under eight years old [1]. Therefore, treatment of scoliosis in young children is very important. Traditional spinal fusion in early childhood may not successfully prevent future spinal deformity as the child grows and has a negative effect on pulmonary function. The inhibition of thoracic growth results in a smaller chest with a decreased vital capacity [7]. Conservative treatment such as braces or casts has generally been unsuccessful in children with progressive curves.

Fusionless scoliosis surgery is perhaps the only acceptable method to solve the aforementioned problems; these include traditional single and DTGRs, vertical expandable prosthetic titanium rib implant (VEPTR), and magnetically controlled growing rods (MCGR) in modern spinal instrumentation. Fusionless scoliosis surgery was first described by Harrington et al. in the 1960s, using a single hook proximally and distally with periodic rod lengthening [8]. Then Luque trolleys, rods with wire loops as internal fixation, with or without a short apical fusion or convex hemiepiphysiodesis were used with successful results but high complication rates [9-11]. Generally, complications were related to wire and rod breakage, as well as auto fusion of the spine.

The single TGR technique has been widely used in previous studies, although complications including implant-related events such as rod breakages and hook displacements, difficult spinal balance control, and unstable fixation have been recorded with this technique [12,13]. Akbarnia and Thompson popularized the DTGR technique [3,5,14]. With this technique, the results of initial correction, maintenance of correction, and spinal growth per year were better with dual rods and complication rates decreased [5,15].

In the current study, the DTGR technique was used because of its superiority to single rod. The modification in this technique that was applied to the patients in this study was the use of pedicle screws as anchors in both the proximal and distal foundations. Mahar et al. showed that a foundation composed of four pedicle screws implanted in two adjacent vertebral bodies provides the strongest construct in pullout testing, and both screw-screw constructs were statistically stronger than either construct containing hooks [4]. In the current study, the use of pedicle screws was seen to be an effective and safe technique that helped to achieve initial correction and maintenance of the correction with few complications. No cross-links were used, as Mahar showed that a cross-link does not enhance fixation if pedicle screws are used [4]. In the current study, correction in the major Cobb angle was 57.9% at the postinitial period and 69.5% at the final follow-up. This correction rate, especially the maintenance of correction up to the final follow-up was more successful than the rates reported in many studies in the literature [3,16,17]. This can be considered to have been due to two important factors; using only pedicle screws as anchors and more frequent lengthenings. With more frequent lengthenings, the continued growth of the spine per year after the initial procedure equaled or surpassed the normal growth of the spine because of the effect of distraction on immature vertebral growth [5]. Olgun et al. reported that growth within the instrumented segment was higher than the growth outside the instrumented part of the vertebrae [18]. The current study experience was similar to that study. The time between two lengthenings was 6.9 months in the current study, and the mean T1-S1 length increase was 1.78 cm per year. In a study by Akbarnia of 13 scoliosis children treated with DGRs, the interval of lengthening was 9.4 months and the T1-S1 length increase was reported to be 1.46 cm per year [5]. Those lengthened at or less than six months had a higher annual growth rate of 1.8 cm versus 1.0 cm, and significantly greater scoliosis correction (79% versus 48%) than those lengthened less frequently. Sankar reported 1.76 cm per year length increase in T1-S1 measurement with a 6.8-month lengthening interval [16].

One of the most used predictors of lung function is the SAL index. In the current study, the mean ratio for SAL increased from 0.885 to 0.956 postinitially and to 0.985 at the final follow-up. Elsebai reported that SAL increased from 0.81 to 0.94 at the final follow-up and Wang reported an improvement of SAL from 0.84 to 0.96 [19,20]. The SAL ratio improvement degree in the study is comparable to that of other studies in the literature.

In the current study, a statistically significant change was determined in the coronal balance between the preinitial and postinitial periods and between the preoperative measurement and the final follow-up (p<0.05). However, no significant change was determined in sagittal balance. Thompson reported no significant change was found in sagittal balance in patients treated with GR due to EOS [14]. Atici et al. also found no statistically significant improvement in sagittal balance and spinopelvic parameters in 23 patients treated with GRs [21]. The authors claimed that the GR technique did not provide a statistically significant improvement in the sagittal spinal parameters except for kyphosis.

Most authors suggest the use of a brace after GR surgeries [17,20,22]. Postoperative bracing was employed in only four patients in our study, two of which were syndromic patients (Campomelic dysplasia, Kabuki Make-Up syndrome) with poor bone quality. As pedicle screws were used for fixation for all foundations, this can be considered to have improved the stability and reduced the necessity for bracing. Not using any brace was also cost-effective and was seen to have an uplifting effect on patients and families during the treatment period. 

During the distraction period, there were a total of 18 (9.6%) complications in 187 procedures: 44.4% of the complications were superficial wound infections and 56.6% were implant-related. No neurological complication or correction loss due to implant-related complications occurred. Akbarnia reported that in 23 patients treated with the DGR technique, 11 patients (47.3%) developed complications, five of which were implant-related. Four patients underwent unplanned surgeries [23]. Thompson reported two patients (29%) with complications in a series of seven patients treated with DGR. The complications were one rod breakage and one hook dislodgement [14]. Upasani et al. reported a total of 263 complications in 87 patients (79%) resulting in 84 unplanned surgeries at an average follow-up period of 8.1 years [24]. The complication rate in the current study was generally lower than the complication rates reported in previous studies. This can be attributed to more stable fixation with pedicle screws and more frequent lengthening resulting in reduced complication rates. To be objective, however, more complications may occur as the follow-up period increases.

The current and more popular technique of growth-sparing surgeries is magnetically controlled growing rod (MCGR) because it requires no repeated planned surgeries for lengthening. The first studies with short-term follow-up confirmed that MCGR was safe and provided adequate distraction similar to GRs and no major complications were observed [25,26]. However, in more recent studies, the medium-term results are not as promising as the previously reported early results. Teoh et al. reported that six of eight (75%) patients treated with MCGR required revision surgery during a four-year follow-up period [27]. These were due to four rod problems, three proximal screw pull-outs, and the development of proximal junction kyphosis. Recently, Cheung et al. reported the 6.1-year follow-up results of MCGR; it was stated that correction of Cobb angle and length gained by distractions were successful, however, rates of complications and implant failure with proximal foundation problems were higher than expected [28]. Compared with TGRs, MCGR has a lower infection rate but does not appear to prevent implant-related complications [29,30]. Longer follow-up and larger groups of patients are needed to determine the outcomes of this new technique.

## Conclusions

The TDGR technique with proximal and distal pedicle screws as anchors is a safe and effective treatment for deformity control in selected patients with EOS. The application of pedicle screws and routine lengthening performed every six months may decrease the rates previously reported of implant-related problems. Furthermore, acceptable improvements in the deformity, spinal growth, SAL, and coronal balance could be achieved with this technique. At the same time, it does not have any negative effects on the sagittal balance. The most negative feature of this traditional technique is the need for repeated surgical interventions and the complications that may occur as a result.
